# Comparison of the efficacy of MOE and PMO modifications of systemic antisense oligonucleotides in a severe SMA mouse model

**DOI:** 10.1093/nar/gkaa126

**Published:** 2020-02-27

**Authors:** Lei Sheng, Frank Rigo, C Frank Bennett, Adrian R Krainer, Yimin Hua

**Affiliations:** 1 Jiangsu Key Laboratory for Molecular and Medical Biotechnology, College of Life Sciences, Nanjing Normal University, Nanjing 210023, China; 2 Department of Orthopedics, the Second Affiliated Hospital of Soochow University, Suzhou, Jiangsu 215004, China; 3 Cold Spring Harbor Laboratory, PO Box 100, Cold Spring Harbor, New York, NY 11724, USA; 4 Ionis Pharmaceuticals, Carlsbad, CA 92010, USA; 5 Institute of Neuroscience, Soochow University, 199 Ren-Ai Road, Suzhou, Jiangsu 215123, China

## Abstract

Spinal muscular atrophy (SMA) is a motor neuron disease. Nusinersen, a splice-switching antisense oligonucleotide (ASO), was the first approved drug to treat SMA. Based on prior preclinical studies, both 2′-*O*-methoxyethyl (MOE) with a phosphorothioate backbone and morpholino with a phosphorodiamidate backbone—with the same or extended target sequence as nusinersen—displayed efficient rescue of SMA mouse models. Here, we compared the therapeutic efficacy of these two modification chemistries in rescue of a severe mouse model using ASO10-29—a 2-nt longer version of nusinersen—via subcutaneous injection. Although both chemistries efficiently corrected *SMN2* splicing in various tissues, restored motor function and improved the integrity of neuromuscular junctions, MOE-modified ASO10-29 (MOE10-29) was more efficacious than morpholino-modified ASO10-29 (PMO10-29) at the same molar dose, as seen by longer survival, greater body-weight gain and better preservation of motor neurons. Time-course analysis revealed that MOE10-29 had more persistent effects than PMO10-29. On the other hand, PMO10-29 appears to more readily cross an immature blood-brain barrier following systemic administration, showing more robust initial effects on *SMN2* exon 7 inclusion, but less persistence in the central nervous system. We conclude that both modifications can be effective as splice-switching ASOs in the context of SMA and potentially other diseases, and discuss the advantages and disadvantages of each.

## INTRODUCTION

Antisense oligonucleotides or oligomers (ASOs) are short synthetic nucleic acid analogs, which bind RNA targets through Watson–Crick base pairing, resulting in reduced gene expression or alterations in RNA processing, depending on their design. Natural nucleic acids are rapidly degraded by endogenous nucleases *in vivo*. Therefore, it is important to chemically modify oligonucleotides to increase their resistance to various nucleases, as well as their binding affinity to RNA targets (1,2). Phosphorothioate (PS) is a first-generation modification, in which a non-bridging oxygen is replaced by a sulfur atom in the phosphate backbone. Although PS modification significantly increases ASO half-life *in vivo*, reflecting increased protein binding and increased resistance to nuclease cleavage, it reduces the affinity for the target RNA; moreover, PS-modified DNA oligonucleotides support RNase H activity (1,2). Second-generation modifications, such as 2′-*O*-methyl (2′-*O*-Me), 2′-*O*-methoxyethyl (MOE) (3), constrained ethyl (cEt) (4), and locked nucleic acid (5) further increase metabolic stability of ASOs, as well as the binding affinity for RNA *in vivo*, conferring enhanced drug-like properties. Phosphordiamidate (PDA) morpholino oligomers (PMOs) replace the pentose sugar with a morpholine ring, and the phosphate with a neutral PDA linkage. PMOs exhibit similar binding affinity to RNA as DNA, have significantly enhanced metabolic stability as well as low protein binding due to the uncharged backbone, and do not support RNase H activity (6,7). The low protein binding feature also makes high-dose PMOs safer *in vivo*, compared to other chemistries.

In recent years, ASOs have emerged as a new paradigm for drug development to treat human diseases. Five ASO drugs have been approved by the U.S. Food and Drug Administration (FDA): fomivirsen, mipomersen, eteplirsen, nusinersen and inotersen (8–12). Notably, in 2016, two ASOs were approved to treat neuromuscular diseases: eteplirsen, a morpholino oligomer that was conditionally approved in the US to treat Duchenne muscular dystrophy (DMD), and nusinersen, an MOE/PS-modified ASO, which was approved in the US, and subsequently in Europe and other areas, to treat spinal muscular atrophy (SMA) (9–11,13). Both drugs work through redirecting pre-mRNA splicing, but in opposite directions: eteplirsen restores the reading frame of the *DMD* gene that carries various frame-shifting mutations or deletions, by promoting exon 51 skipping (13), whereas nusinersen increases expression of functional SMN protein from the *SMN2* gene by promoting exon 7 inclusion (14,15).

SMA is a fatal genetic disorder that manifests during infancy or childhood, with an incidence of approximately one in 10 000 live births (16,17). It is caused by deletion or mutation of *survival of motor neuron 1* (*SMN1*), a gene that encodes the essential and ubiquitously expressed protein SMN. Low levels of this protein result in degeneration of spinal-cord motor neurons and consequent atrophy of skeletal muscles (18). All animal species possess only one *Smn* gene, and the homozygous knockout is embryonic lethal; humans, however, have an additional *SMN2* gene that expresses the same SMN protein, albeit in low amounts (18,19). Compared to *SMN1*, there are two key nucleotide transitions in *SMN2*—C6T in exon 7, and to a lesser degree G-44A in intron 6—which cause skipping of exon 7 in approximately 90% of *SMN2* transcripts (20,21). The resulting SMNΔ7 protein isoform is unstable and rapidly degraded. The small amount of full-length (FL) protein expressed by *SMN2* is not sufficient to compensate for the loss of *SMN1*. SMA patients have two or more copies of *SMN2*, and the copy number is inversely correlated with the age of disease onset and its severity (22). Nusinersen (previously called ASO10-27), is 18-nt long, and prevents the binding of hnRNP A1/A2 repressor proteins to a splicing silencer (ISS-N1) near the 5′ splice site of *SMN2* intron 7, thereby efficiently correcting *SMN2* splicing *in vivo* (23–28).

Clinical trials demonstrated the effectiveness of MOE/PS-modified nusinersen (29–31). PMOs that target the same ISS-N1 region showed marked efficacy in SMA mouse models, correcting *SMN2* splicing, ameliorating symptoms and extending the lifespan (32,33). We previously showed that nusinersen is more potent than morpholino/PDA-modified ASO10-29—a 2-nt longer version—in correcting *SMN2* splicing in the central nervous system (CNS) of an SMA mouse model after ICV bolus injection, likely due to enhanced accumulation and/or slower clearance of nusinersen in this compartment (34). However, to date, no thorough head-to-head comparison of the efficacy of the two modifications with respect to antisense splicing therapy in SMA mouse models has been performed, and therefore, it is unclear if one of the modifications might be more advantageous for SMA therapy. Published studies do not allow for a direct comparison, because the ASOs employed differ not only in chemistry, but also in length, administration route and dose, and different mouse models were used. Addressing this issue may help design or optimize ASO drugs for treating SMA as well as other diseases.

In this study, we compared the efficacy of MOE/PS and morpholino/PDA chemistries using ASO10-29 in the severe Taiwanese SMA mouse model; we refer to these two ASOs as MOE10-29 and PMO10-29, respectively. Considering that peripheral delivery of nusinersen reaches the CNS before postnatal maturation of the murine blood-brain barrier (BBB) and strongly rescues a severe SMA mouse model (25,26), we focused on subcutaneous (SC) administration in neonate mice and comprehensively assessed the effects of the two ASOs on *SMN2* splicing in various tissues and on phenotypic rescue. The two ASOs were delivered at early postnatal days at the same molar doses. We found that MOE10-29 outperformed PMO10-29 in improving the phenotype, including a more robust survival increase. This result is consistent with MOE10-29’s more persistent effects on *SMN2* splicing in all peripheral tissues, which indicate that MOE10-29 has a longer pharmacodynamic half-life than PMO10-29, though the initial effect of PMO10-29 on *SMN2* splicing is similar or slightly better. On the other hand, PMO10-29 appears to more readily cross the immature BBB into the CNS, based on splicing analysis; however, its effect in the CNS is short-lived. We conclude that both MOE-PS and morpholino chemistries are effective for splice-switching ASOs to treat SMA mice, though the latter may require higher doses or more frequent dosing to achieve similar phenotypic rescue.

## MATERIALS AND METHODS

### Oligonucleotide synthesis

The synthesis and purification of MOE10–29 (5′-ATTCACTTTCATAATGCTGG-3′) with PS backbone and 5-methyl cytosines were performed as described (24). PMO10-29 (5′-ATTCACTTTCATAATGCTGG-3′) was purchased from Gene Tools (Philomath, OR, USA). All oligonucleotides were dissolved in 0.9% saline.

### Animals and drug delivery

All mouse protocols were in accordance with Cold Spring Harbor Laboratory's Institutional Animal Care and Use Committee guidelines. The initial breeding pairs of human *SMN2* transgenic mice were purchased from Jackson Laboratory (stock number 005058), and were originally developed by Hsieh-Li *et al.* (35). The severe SMA model (*Smn*^−/−^; *SMN2*^2TG/0^) was generated as previously described (25). The oligonucleotide solutions were injected subcutaneously into the upper back with a 5-μl syringe and a 33-gauge custom removable needle (Hamilton).

### Radioactive RT-PCR

Mouse tissues were pulverized in liquid N_2_ with mortar and pestle, and total RNA was extracted with Trizol (Invitrogen). One microgram of total RNA was reverse-transcribed with ImProm-II Reverse Transcriptase (Invitrogen). For radioactive RT–PCR, the human-specific primer pair E4-33to55-F and E8-15to36-R was used for amplifying human *SMN2* transcripts in RNA samples from mouse tissues, as described (25); all PCR products were labeled with α-^32^P-dCTP and analyzed by 6% native polyacrylamide gels, followed by phosphorimage analysis. The extent of exon 7 inclusion was calculated as described (25), and the signal intensity of each cDNA band was normalized according to its G+C content.

### Western blotting

Twenty milligrams of each mouse tissue was pulverized in liquid N_2_ and homogenized in 0.4 ml of protein sample buffer containing 2% (w/v) sodium dodecyl sulphate (SDS), 10% (v/v) glycerol, 50 mM Tris–HCl (pH 6.8) and 0.1 M DTT. Protein samples were separated by 12% SDS-polyacrylamide gel electrophoresis and electro-blotted onto nitrocellulose membranes. The membranes were blocked for 2 h with 5% (w/v) non-fat milk in Tris-buffered saline containing 0.05% Tween-20 (TBST), and then incubated overnight at 4°C with primary antibodies: monoclonal anti-SMN (BD Biosciences, 1:500) and anti-α-tubulin (Sigma). After washing with TBST for three times, the membranes were incubated with secondary IRDye 700CW-conjugated goat anti-mouse or anti-rabbit antibody. Protein signals were detected with an Odyssey instrument (LI-COR Biosciences).

### Histology

Spinal cords were fixed with 4% (v/v) formaldehyde in phosphate-buffered saline overnight. For gem counting in motor neurons and motor-neuron counting in lumbar spinal-cord segments L1–L2, paraffin-embedded 6-mm sections were treated with citrate buffer for antigen retrieval, and incubated with goat anti-ChAT antibody (Millipore) and/or mouse SMN antibody (BD Bioscience) followed by donkey anti-goat Alexa fluor 568 (Invitrogen) and/or donkey anti-mouse Alexa fluor 488 secondary antibodies. For neuromuscular junction (NMJ) staining, mice were anaesthetized by intraperitoneal injection of Nembutal (sodium pentobarbital; 50 mg/kg) or ketamine/xylazine (100 mg/kg ketamine/10 mg xylazine) and transcardially perfused with PBS and then 4% paraformaldehyde. After 24 h post-fixing, the tibialis anterior (TA) and flexor digitorum brevis 2/3 (FDB-2/3) muscles were dissected and teased into layers 5–10 fibers thick to facilitate penetration of antibodies, including anti-neurofilament (1:2000; Chemicon) and anti-synaptophysin (1:200, Invitrogen) antibodies. Acetylcholine receptors (AChRs) were labeled with Alexa Fluor 555-conjugated α-bungarotoxin (α-BTX, Invitrogen). Confocal immunofluorescence imaging was done with an LSM710 confocal microscope (Carl Zeiss) by merging Z-stacks of multiple planes into one image.

### Motor-function tests

Grip strength was measured using a grip-strength meter (Columbus Instruments) as described (26). Rotarod tests were carried out with a RotaRodIV instrument (AccuScan); the rotation was set at a constant rate of 5 rpm in one direction for a maximum of 180 s. Among five trials for each mouse, the longest time that the mouse stayed on the rod was recorded.

### Statistical analysis

Differences between sets of data were analyzed by two-tailed Student's t test using SPSS16.0 (IBM); a value of *P* < 0.05 was considered statistically significant. In all of the figures, data are expressed as mean ± SEM.

## RESULTS

### Systemically administered MOE10-29 outperforms PMO10-29 in improving the phenotype of SMA mice

**Figure 1.**
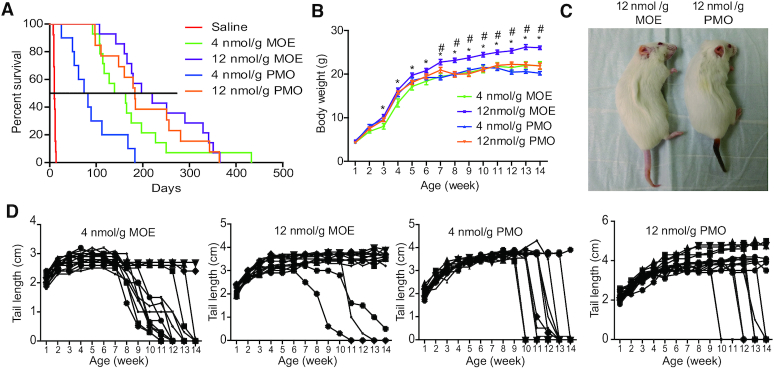
MOE10-29 led to better therapeutic outcomes than PMO10-29 in severe SMA mice. (**A**) Survival curves after two SC injections of saline (*n* = 12), MOE10-29 (4 nmol/g, *n* = 14; 12 nmol/g, *n* = 14) and PMO10-29 (4 nmol/g, *n* = 14; 12 nmol/g, *n* = 14) between P0 and P1. (**B**) Body-weight curves after SC injections of MOE10-29 and PMO10-29 as in (A). (**C**) Representative pictures of high-dose MOE10-29- and PMO10-29- treated SMA mice on P60. (**D**) Tail length was measured weekly for up to 14 weeks (*n* = 12). (*) *P* < 0.05 high dose versus low dose of the same ASO; (^#^) *P* < 0.05 versus all other groups.

To compare the efficacy of MOE10-29 and PMO10-29, we used the severe Taiwanese mouse model, which has a mean survival of 10 days. Efficient rescue of this model requires SMN restoration in peripheral tissues (25,26). Therefore, we delivered these two ASOs by SC injection. Newborn pups were given two injections between postnatal day 0 (P0) and P1 at two doses: 4 and 12 nmol/g/injection, corresponding to 32 and 95 mg/kg/injection respectively for MOE10-29, and 27 and 81 mg/kg/injection, respectively, for PMO10-29. The higher dose was expected to generate robust rescue based on previous studies (25,32). Each group had 12 mice (six female and six male mice). As expected, treatment with either MOE10-29 or PMO10-29 dramatically improved survival and showed dose-dependent effects; however, MOE10-29 had a better therapeutic outcome than PMO10-29. The low dose of MOE10-29 increased the median and mean survival to 139 and 152 days, respectively, compared with 89 and 98 days, respectively, for the low dose of PMO10-29 (Figure 1A). In addition, we observed that some low-dose PMO10-29-treated mice suddenly died, without showing any obvious signs of disease morbidity, such as weight loss and tissue necrosis, whereas this only rarely occurred in MOE10-29-treated mice. Similarly, high-dose MOE10-29 improved the median and mean survival to 197 and 233 days, respectively, whereas high-dose PMO10-29 improved the median and mean survival to 183 and 204 days, respectively (Figure 1A). These data indicate that systemic administration of MOE10-29 is more effective in extending the lifespan of SMA mice at the doses tested.

Body weight gain, which is severely impaired in the mouse model, was also monitored weekly for up to 14 weeks. The average body weight of mice in the three groups all plateaued at ∼21 *g* at 11 weeks post-treatment, except for the high-dose MOE10-29 group, in which treated mice reached 26 g in body weight at the 14th week post-treatment, representing a 24% increase compared with the other groups (Figure 1B and C).

Tissue necrosis is a characteristic feature in mild SMA mice, which typically begins to show at the fourth week after birth. Extending the lifespan of severe SMA mice to >3 weeks by drug treatment allows necrosis to appear, and the poorer the rescue, the more severe the necrosis. Necrosis generally occurs in the tail and ear pinnae, but can also extend to the nose, limbs and anus (25,36). After treatment with either MOE10-29 or PMO10-29 at either dose, all mice exhibited tail necrosis, and the earliest occurrence was at ∼6 weeks (Figure 1C and D). The tail necrosis in both high-dose groups was much less severe than in the low-dose groups (Figure 1D). Interestingly, the process of necrosis was much slower in MOE10-29-treated mice than in PMO10-29-treated mice. When necrosis started from the tail tip, it took 3–5 weeks for MOE10-29-treated mice to completely lose the tail, whereas it took less than a week, and often just 2–3 days, for PMO10-29-treated mice to lose the tail (Figure 1D). These data indicate that MOE10-29 is more efficacious and appears to have a longer duration of effect in preventing tissue necrosis, compared to PMO10-29.

### ASO effects on exon 7 inclusion in peripheral tissues

**Figure 2.**
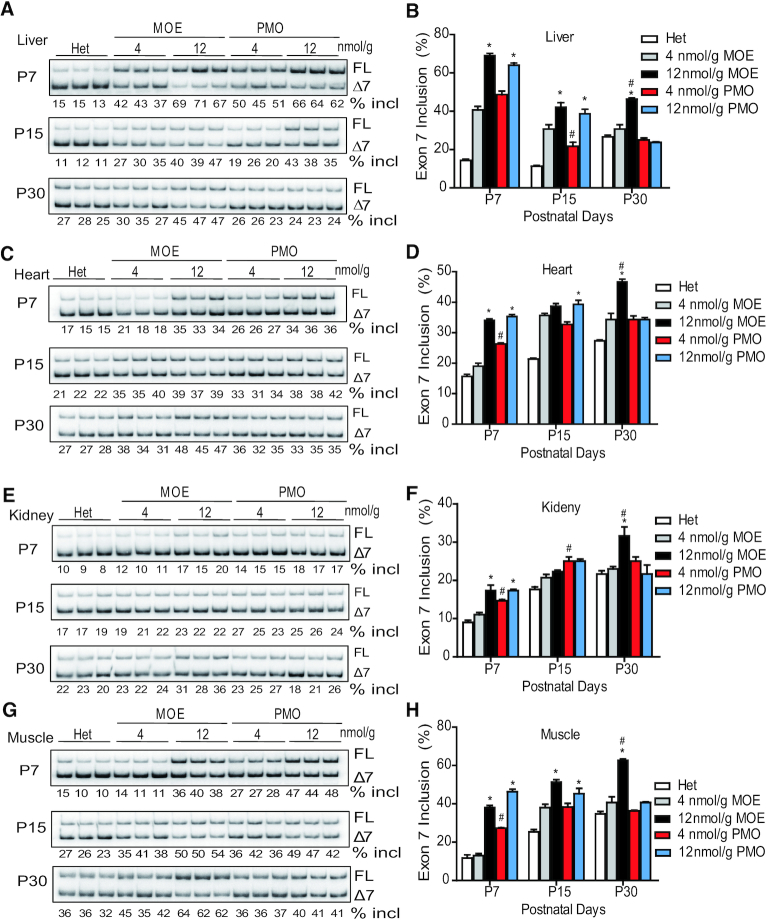
*SMN2* splicing changes in mouse peripheral tissues after SC injection of MOE10-29 or PMO10-29. Splicing analysis of liver (**A** and **B**), heart (**C** and**D**), kidney (**E** and**F**) and muscle (**G** and**H**) tissues from MOE10-29- and PMO10-29-treated mice by radioactive RT-PCR at three time points (P7, P15 and P30) after treatment as in Figure 1 (*n* = 4). (*) *P* < 0.05 high dose versus low dose of the same ASO. % incl, percentage of exon 7 inclusion; (^#^) *P* < 0.05 MOE versus PMO at the same dose.

**Figure 3.**
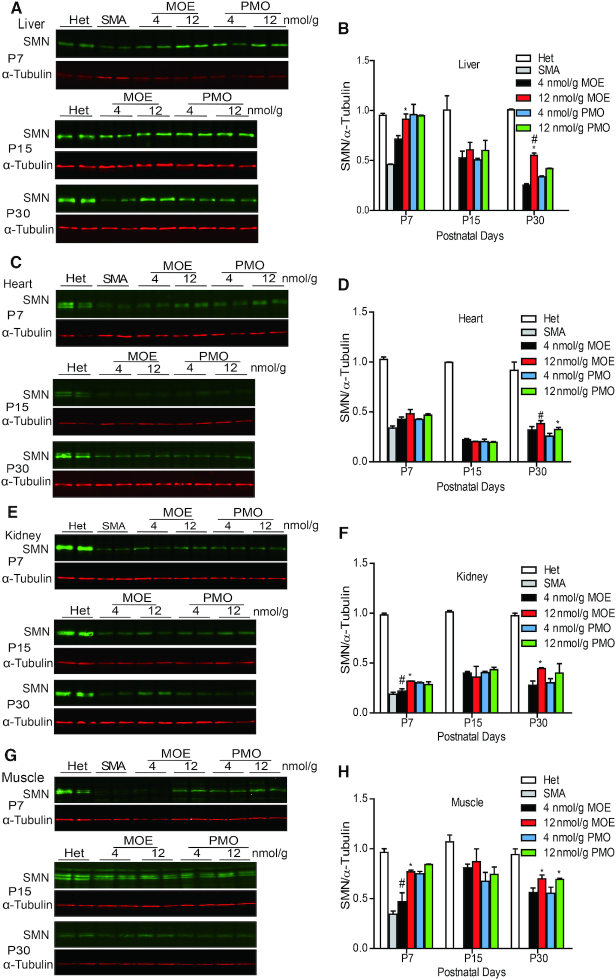
SMN protein expression in mouse peripheral tissues after SC injection of MOE10-29 or PMO10-29. Western blotting analysis of SMN levels in mouse liver (**A** and**B**), heart (**C** and**D**), kidney (**E** and**F**) and muscle (**G** and**H**) tissues at three time points (P7, P15 and P30) after the treatments described in Figure 1 (*n* = 4). (*) *P* < 0.05 high dose versus low dose of the same ASO; (^#^) *P* < 0.05 MOE versus PMO at the same dose.

The differences in therapeutic outcomes after treatment with the two ASOs should reflect their respective capabilities in correcting *SMN2* splicing in affected tissues. Some recent studies showed that multiple peripheral tissues, such as heart and liver, are critical in the pathogenesis of SMA, at least in severe mouse models (25,37–40). We first examined *SMN2* splicing changes in peripheral tissues, including the liver, heart, kidney and muscle, at three time points (P7, P15 and P30) after treatment with four different doses (4, 12, 18 and 21 nmol/g) of the two ASOs (Figure 2 and Supplementary Figure S1). Both ASOs, at all doses, promoted exon 7 inclusion in all examined tissues. The PMO appeared to have a faster onset of action, as its effects in all tissues 7 days after injection were higher than those of the MOE ASO in the low-dose groups (Figure 2 and Supplementary Figure S1). By 15 days, however, their effects became similar. Skeletal muscle was a favorable target tissue for PMO10-29, resulting in stronger splicing correction on P7 at all tested doses, compared to MOE10-29. In the higher-dose groups, the splicing changes on P7 and P15 were similar for the two oligomers. However, by P30, the percentage of exon 7 inclusion in all tissues in the high-dose PMO10-29 group decreased; in contrast, mice in the high-dose MOE10-29 group maintained a higher level of exon 7 inclusion in all examined peripheral tissues, compared to all other groups. These data highlight that the effects of MOE10-29 are more sustained than those of PMO10-29 in these tissues (Figure 2 and Supplementary Figure S2). Interestingly, except for the liver, the percentage of exon 7 inclusion in the heart, skeletal muscles, and kidneys of mice treated with high-dose MOE10-29 increased over time, which is likely due to both the sustained effects of the MOE oligomer, and improvement in *SMN2* splicing with age during the first postnatal month, as seen in untreated heterozygous mice (Figure 2 and Supplementary Figure S3). We also performed western-blotting analysis, and observed the corresponding changes in full-length SMN protein levels in these tissues in ASO-treated mice (Figure 3).

### Systemically delivered PMO10-29 penetrates more readily into the CNS than MOE10-29

**Figure 4.**
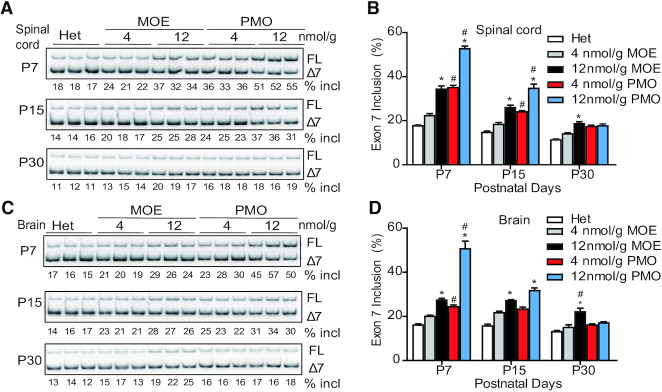
*SMN2* splicing changes in mouse CNS tissues after SC injection of MOE10-29 or PMO10-29. Splicing analysis of spinal cord (**A** and **B**) and brain (**C** and **D**) from MOE10-29- and PMO10-29-treated mice by radioactive RT-PCR at three time points (P7, P15 and P30) after treatment as in Figure 1 (*n* = 4). (*) *P* < 0.05 high dose versus low dose of the same ASO; (^#^) *P* < 0.05 MOE versus PMO at the same dose.

**Figure 5.**
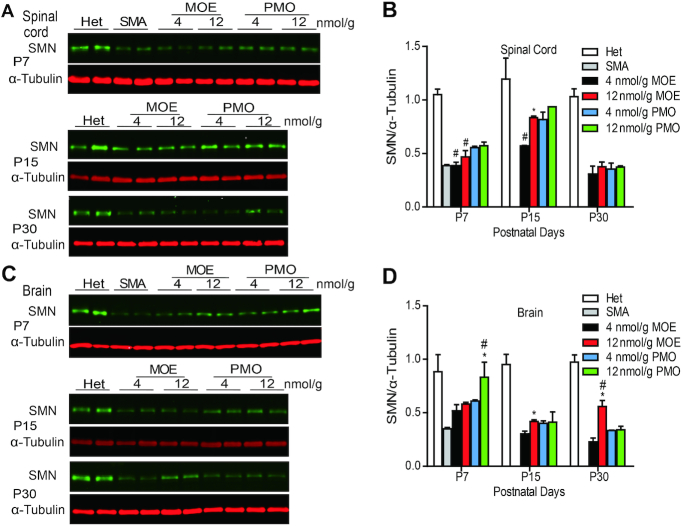
SMN protein expression in mouse CNS tissues after SC injection of MOE10-29 and PMO10-29. Western blotting analysis of SMN levels in mouse spinal cord (**A** and **B**) and brain (**C** and **D**) tissues at three time points (P7, P15 and P30) after treatment as in Figure 1 (*n* = 4). (*) *P* < 0.05 high dose versus low dose of the same ASO; (^#^) *P* < 0.05 MOE versus PMO at the same dose.

Our previous studies showed that systemically administrated ASOs can cross the immature BBB in neonatal mice and reach CNS tissues (25,26). Therefore, we compared the effects of the two ASOs on *SMN2* splicing in the CNS after SC injection. RNA samples from brain and spinal cord tissues were isolated on P7, P15 and P30, after two SC injections of each ASO at 4, 12, 18 or 21 nmol/g between P0-P1. Radioactive RT-PCR was performed to analyze *SMN2* exon 7 inclusion (Figure 4 and Supplementary Figure S1). Interestingly, PMO10-29 treatment enhanced *SMN2* exon 7 inclusion in the CNS on P7 and P15 more robustly than MOE ASO treatment. However, from P7 to P30, the exon 7 inclusion levels in the PMO10-29 groups decreased in the spinal cord (65% for low dose and 82% for high dose) and brain (64% for low dose and 88% for high dose), whereas mice treated with high-dose MOE10-29 showed sustained high levels of exon 7 inclusion (Figure 4 and Supplementary Figure S2), suggesting that MOE10-29 has a longer half-life than PMO10-29 not only in peripheral tissues, but also in the CNS. Immunoblotting revealed similar changes in SMN protein levels in the spinal cord and brain, corresponding to the mRNA level changes in all ASO-treated groups (Figure 5). These results demonstrate that PMO10-29 crosses the immature BBB more readily than MOE10-29, but the effect of MOE10-29 in the CNS lasts longer.

### Gem counts in the spinal cord

**Figure 6.**
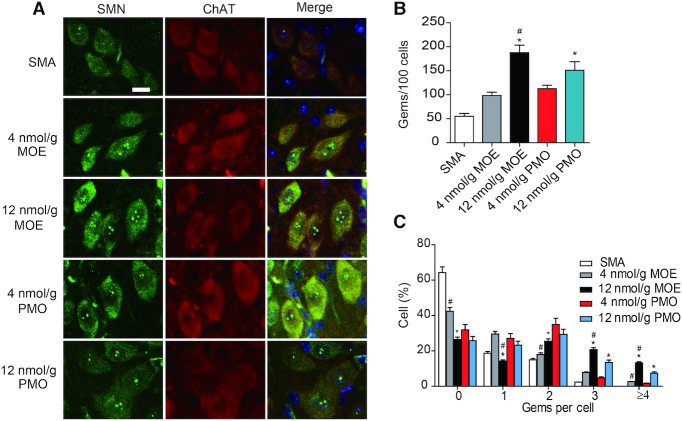
Gem counts in motor neurons of spinal cord segments L1–L2 obtained from ASO-treated SMA mice. (**A**) SMA mice were SC injected twice with saline (*n* = 4), MOE10-29 (4 nmol/g, *n* = 4; 12 nmol/g, *n* = 4) and PMO10-29 (4 nmol/g, *n* = 4; 12 nmol/g, *n* = 4) between P0 and P1. Tissues were collected on P9. Gems and motor neurons were labeled with SMN and ChAT; nuclei were counterstained with DAPI. (**B**) Quantitation of gems per 100 motor neurons from (A). (**C**) Percentages of motor neurons containing 0, 1, 2, 3, or ≥4 gems from A. (*) *P* < 0.05 high dose versus low dose of the same ASO; (^#^) *P* < 0.05 MOE versus PMO at the same dose.

SMN protein is localized in the cytoplasm and in a subnuclear structure termed gemini of Cajal body, or gem (41). Gem numbers positively correlate with cellular SMN levels (42). Therefore, we examined gem numbers in spinal-cord motor neurons on P9, after ASO treatment as above. A monoclonal anti-SMN antibody was used to detect SMN in the ventral horn of lumbar segments L1–L2, with choline acetyltransferase (ChAT) being used to label motor neurons. As shown in Figure 6, both MOE10-29- and PMO10-29-treated SMA mice had more gems in motor neurons than untreated SMA mice, with increases of 1.8- (low MOE), 3.4- (high MOE), 2.0- (low PMO) and 2.7-fold (high PMO), respectively. This was reflected by increases in both the number of cells with gems and the number of gems per cell (Figure 6C). Paradoxically, in spite of the higher SMN protein levels in the spinal cord of PMO10-29-treated mice, the number of gems in motor neurons in the high-dose MOE group was markedly higher than that in the high-dose PMO10-29 group (Figure 6). The cause of this apparent discrepancy is currently unknown, but may reflect differences in the uptake of ASOs with each chemistry in different cell types.

### Spinal-cord motor-neuron counts and motor-function tests

**Figure 7.**
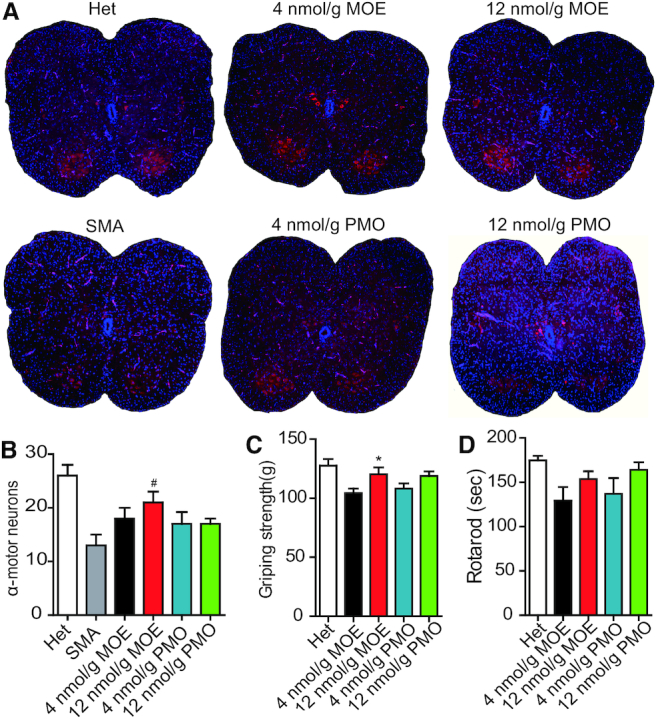
Motor-neuron counts in spinal cord segments L1–L2 and motor-function tests of SMA mice. (**A**) SMA mice were treated with saline (*n* = 4), MOE10-29 (4 nmol/g, *n* = 4; 12 nmol/g, *n* = 4) and PMO10-29 (4 nmol/g, *n* = 4; 12 nmol/g, *n* = 4) as described in Figure 6. Untreated heterozygous mice (Het, *n* = 13) were used as normal controls. Tissues were collected on P9. Motor neurons and nuclei were labeled with ChAT and DAPI. (**B**) Motor-neuron number was calculated based on (A) (four mice per group and three counts per mouse). (**C** and **D**) Grip strength and rotarod tests of rescued SMA mice (*n* = 12) at 3 months of age. (*) *P* < 0.05 high dose versus low dose of the same ASO; (#) *P* < 0.05 MOE versus PMO at the same dose.

Motor-neuron degeneration is the hallmark of SMA. To count motor neurons, we used ChAT staining to label the cells in sections of lumbar segments L1–L2 derived from SMA mice on P9, after treatment with MOE10-29, PMO10-29 or saline control. As expected, untreated (saline control) SMA mice had a strikingly low number of motor neurons in the spinal cord, compared to heterozygotes, with a reduction of 50% (Figure 7A). In contrast, mice treated with ASOs showed a marked increase in the number of ChAT-positive cells, with an increment of 38% (low MOE), 62% (high MOE), 31% (low PMO) and 31% (high PMO), respectively (Figure 7A and B). These results indicate that MOE10-29 is more effective in rescuing motor neurons. The marked increase in motor-neuron counts in high-dose-MOE10-29-treated mice over high-dose-PMO-treated mice is consistent with the above gem-counting data, supporting the notion that motor neurons may favor MOE/PS modification over morpholino/PDA, in terms of spontaneous ASO uptake. We next measured motor function in ASO-treated mice using grip-strength and rotarod tests at 3 months old. There were no significant differences in grip strength and rotarod tests in mice treated with the same dose of MOE10-29 and PMO10-29 (Figure 7C and D). Some low-dose-ASO treated mice died within 24 h after the behavioral experiments.

### Structural analysis of the neuromuscular junction (NMJ)

**Figure 8.**
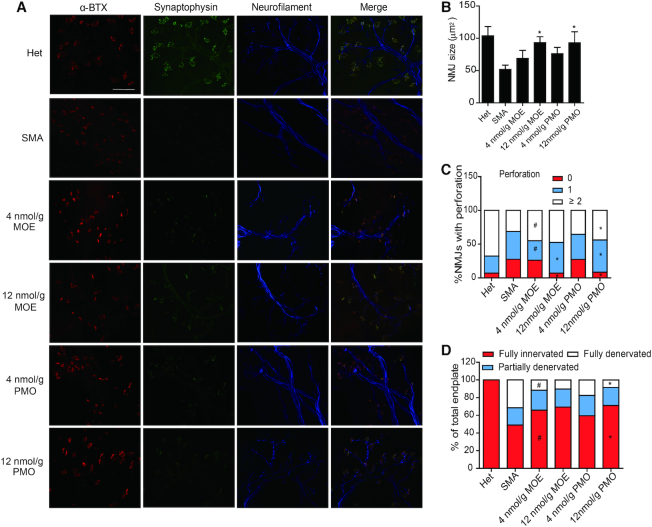
NMJ staining of the flexor digitorum brevis 2 and 3 (FDB-2/3) muscles of SMA mice. (**A**) SMA mice were treated with saline (*n* = 4), MOE10-29 (4 nmol/g, *n* = 4; 12 nmol/g, *n* = 4) and PMO10-29 (4 nmol/g, *n* = 4; 12 nmol/g, *n* = 4) as in Figure 6. Untreated heterozygous mice (Het, *n* = 4) were used as normal controls. FDB-2/3 were collected on P9 and stained for neurofilament with anti-neurofilament (blue), nerve terminals with anti-synaptophysin (green) and motor endplates with α-bungarotoxin (α-BTX, red). (**B**) NMJ area was measured from (A) (four mice per group and three counts per mouse). (**C**) Quantification of perforations identified by α-BTX, based on (A) (4 mice per group and 3 counts per mouse). (**D**) Percentages of innervated endplates (red), partially denervated endplates (blue), and fully denervated endplates (white) were quantitated based on (A) (four mice per group and three counts per mouse). (*) *P* < 0.05 high dose versus low dose of the same ASO; (^#^) *P* < 0.05 MOE versus PMO at the same dose.

Defects in NMJs, such as denervation, immature endplates, and poor terminal arborization are typical in SMA mouse models. Earlier studies showed that severe denervation occurs selectively in axial and appendicular muscles, and FDB-2/3 are the most severely affected muscles in SMA mouse models (43,44). We examined NMJ structures in FDB-2/3 and TA muscles on P9. We used anti-synaptophysin and anti-neurofilament antibodies to label presynaptic nerve terminals, and α-bungarotoxin to label postsynaptic endplates. The extent to which the NMJ pathology can be rescued by ASO treatment is an important parameter to evaluate the efficacy of ASO drugs. Similar to what was previously reported, SMA mice displayed smaller endplate sizes and less complexity (reflected by the number of perforations per NMJ), in comparison to heterozygous mice (Figure 8 and Supplementary Figure S4). After treatment with MOE10-29 or PMO10-29, the endplate size in FDB-2/3 muscles increased by 33% (low MOE), 80% (high MOE), 46% (low PMO) and 80% (high PMO), respectively, and the percentage of NMJs containing ≥ 2 perforations increased by 44, 52, 13 and 40%, respectively, compared to severe SMA mice (Figure 8B and C).

Denervated NMJs were defined as α-BTX-labeled endplates without overlapping synaptophysin-stained axons, and partial denervation was defined as ≤50% occupancy of presynaptic nerve terminals in an endplate. After ASO treatment, the percentage of fully and partially denervated NMJs in FDB-2/3 muscles decreased by 35% (low MOE), 42% (high MOE), 22% (low PMO) and 45% (high PMO), compared to untreated mice (Figure 8A and D). There was also a marked NMJ improvement, similar between the two ASO treatments, in TA muscles (Supplementary Figure S4). Based on the immunostaining analysis of NMJs, we conclude that both ASOs have similar efficacy in rescuing the NMJ pathologies of SMA mice. The lack of differences between the two ASOs in NMJ recovery may reflect the early time point (P9) selected for the analysis.

## DISCUSSION

MOE and morpholino are two chemistries that are frequently used to modify ASOs for clinical applications (45–47). Nusinersen, the first approved therapeutic for SMA, uses the MOE/PS modification and demonstrated robust efficacy in both preclinical and clinical studies (25–26,29–31). PMOs also showed high effectiveness in preclinical SMA mouse models (32,33). In the present study, we thoroughly compared these two chemical modifications in the context of identical-sequence 20-mer ASOs, in a severe SMA mouse model.

### Antisense effect of MOE10-29 is more persistent

By measuring *SMN2* splicing, we found that although PMO10-29 initially displayed a robust effect in promoting exon 7 inclusion in all tested tissues, which was similar to, or on occasion stronger than that of MOE10-29, its effects were not as long-lasting as those of MOE10-29. The fact that MOE10-29 has a longer half-life *in vivo* than PMO10-29 is likely the main reason why MOE10-29 is therapeutically more effective. Our conclusion is consistent with and extends our previous work, which showed that ICV bolus injection of PMO10-29 resulted in less accumulation in CNS tissues compared to nusinersen (ASO10-27) (34).

The approval of nusinersen to treat SMA was a major milestone in the history of oligonucleotide therapeutics, which is becoming an important platform for new drug discovery. The resurgence of oligonucleotide therapeutics is largely attributable to advances in chemical modifications. However, there have been few comparative studies of ASO-modifying chemistries. Nearly two decades ago, Sazani *et al.* developed an elegant EGFP-transgenic mouse model to assay for splice-switching antisense activity with several chemical modifications, including MOE/PS and PMO (48). EGFP is expressed in this model only when an effective ASO is administered to block inclusion of a pseudo-exon in a chimeric minigene comprising the EGFP coding sequence interrupted by a mutated intron 2 of human *HBB* (β-globin) (48,49). The authors examined EGFP expression one day after intraperitoneal injection of ASOs for four consecutive days at 50 mg/kg/day; based on the EGFP readout, MOE/PS had similarly high antisense activity in some tissues as PMO, while outperforming PMO in other tissues; overall, our results are consistent with these data, demonstrating that both modifications can be employed for developing therapeutic ASOs (48). Recently, Prakash *et al.* also compared MOE10-29 versus PMO10-29 in the liver of transgenic SMA mice, and found that both modifications had similar effects in correcting *SMN2* splicing (50), which is also consistent with our data (Figure 2). However, the previous studies did not examine the duration of the effects, a key parameter in drug development. In addition, the comparisons in previous studies all used the same mass doses, instead of molar doses and morpholino-modified ASOs have ∼15% lower molecular weights; thus, the effects of morpholino-modified ASOs were slightly over-estimated.

### Unique features of each modification

One interesting observation about PMO10-29 is its consistent superiority over MOE10-29 in antisense activity in skeletal muscle in the first week after systemic injection (Figure 2 and Supplementary Figure S1). This observation suggests that morpholino-modified ASOs are more readily taken up by skeletal muscle fibers. This feature may give morpholino ASOs an edge for treating skeletal-muscle disorders. On the other hand, the charge-neutral and hydrophobic nature, poor protein binding and lower molecular weight of PMO10-29 may explain why it crosses an immature BBB of neonatal mice more readily than MOE10-29, as shown by *SMN2* splicing in the first two weeks post-treatment (Figure 4). However, this feature of morpholino may be of little significance in terms of ASO therapy, as neither MOE nor morpholino ASOs, typically 18–20mer long, can cross an intact BBB in older animals.

Interestingly, although systemic PMO10-29 resulted in greater CNS penetration, the gem counts on P9 revealed more gems in motor neurons from MOE10-29-treated mice. One explanation for this observation is that motor neurons in particular may favor internalization of ASOs with MOE/PS chemistry over PMO. Alternatively, the PS chemistry itself may play a role in the increase in gem numbers in motor neurons of MOE10-29-treated mice. Recent studies revealed that PS-modified ASOs induce the formation of some cytoplasmic and subnuclear non-membranous structures through binding to their protein components (51,52). Further experiments will be necessary to clarify the mechanism, which may be of therapeutic relevance.

### Therapeutic outcomes and caveats to this comparative study

Administration routes have a big impact on drug efficacy. We previously showed that systemic delivery of nusinersen via SC or intraperitoneal injection robustly rescued SMA mouse models, whereas ICV delivery displayed moderate rescue, indicating that peripheral defects are critical in the mouse models (25,26). Perhaps due to SMN restoration in the CNS being less important in the mouse model, we did not observe significant differences in rescue, particularly of the mean survival, between the two modifications when ASOs were injected ICV (Supplementary Figure S5). Yet all PMO10-29-treated mice survived no longer than 86 days, whereas three MOE10-29-treated mice survived over 86 days (98, 149 and 255 days, respectively), consistent with our conclusion that MOE ASOs have more sustained activities. However, the therapeutic outcomes by ICV-injected ASOs cannot be solely explained by their effects in the CNS, because ASOs are gradually cleared from the CNS into the blood and distributed to peripheral tissues. Porensky *et al.* demonstrated that 8 nmol/g PMO10-29 delivered ICV increased the full-length *SMN2* transcript 2-fold in the liver (32); therefore, excessive doses of PMO in the CNS are safe, but phenotypic improvement is likely dominated by peripheral effects. A different animal model that has CNS-restricted defects would be useful to precisely compare chemical modifications for ASO applications in the CNS.

In this study, we focused on comparing the therapeutic outcome by systemic delivery of the two ASOs, and found that at equivalent concentrations, MOE10-29-treated mice displayed a stronger phenotypic improvement than PMO10-29, including greater body-weight gain, slower necrosis of distal tissues, higher motor-neuron counts and importantly, longer survival. Therefore, we conclude that at the same molar dose, MOE/PS is a more effective chemistry for therapy in a severe SMA mouse model.

On the other hand, the initial effect of PMO10-29 on *SMN2* splicing in all tissues is no less than that of MOE10-29, suggesting that more frequent dosing with PMO10-29 may achieve comparable if not better therapeutic outcome, compared to the two-injection dosing schedule with MOE10-29. In theory, each ASO should be dosed at intervals optimally suited to its respective pharmacokinetics. However, from a clinical perspective, nusinersen is given by lumbar puncture, which is burdensome, and it would be desirable to reduce the dosing frequency, rather than increase it. Indeed, a new clinical trial, DEVOTE, is testing higher nusinersen doses, in part with the hope that fewer lumbar punctures will be needed. Thus, a more realistic strategy would be to increase the dose per injection.

One important aspect in drug development is the toxicity issue. We previously reported significant toxicity of nusinersen after one ICV injection of >15 μg in newborn SMA mice, whereas PMO10-29 displayed no toxicity after one ICV injection of 81 μg (27,32). On the other hand, we did not observe any toxicity when nusinersen was ICV-infused into the CNS of adult mice at 150 μg/day for a week (53). Currently, nusinersen is used clinically at 12 mg per intrathecal injection, four times in the first 2 months, and every 4 months thereafter, and no drug-related toxicities have been observed, although some patients have been treated continuously for up to 7 years. PMOs are less toxic due to their chemical properties and more rapid excretion. It is possible or even likely that comparable efficacy can be achieved with higher doses of PMO, but this would add to the costs of manufacture and treatment. We do believe that PMOs, considering their unique features, may be a good choice to treat certain diseases.

To date, at least six chemistries have been tested to modify ASO10-27 or its shorter or longer versions for therapeutic potential. 2′-F-modified ASO10-27 inhibits *SMN2* splicing, due to recruitment of IFL2 and IFL3 near the 5′ splice site of intron 7 by the 2′-F/RNA duplex (54), resulting in exon 7 skipping, and therefore, this modification cannot be used to treat SMA. 2′-O-Me-modified ASOs efficiently correct *SMN2* splicing in cultured cells, but they are ineffective in mouse models and cause CNS inflammation when centrally delivered (28,34,53,55). Robin *et al.* recently reported a tricyclo-DNA (tcDNA) ISS-N1-targeting 15mer ASO that corrected *SMN2* splicing and halted tissue necrosis in a mild mouse model (56). However, the tcDNA ASO has yet to be further characterized and tested in more severe models. We previously assessed a cEt version of nusinersen, which carries mixed MOE/cEt modifications with a melting temperature 15°C higher than nusinersen, in SMA fibroblasts and *SMN2*-transgenic mice; both ASOs had similar potency for *SMN2* splicing correction, suggesting that cEt also has therapeutic potential, but further increase in the affinity of nusinersen for binding to its target did not increase its potency (34). MOE and morpholino are the only two modifications of ASOs that have been tested in multiple mouse models, including mild and severe ones and showed high efficacy in *SMN2* splicing correction and phenotypic rescue.

Here, our rigorous comparative study with the two ASOs at the same molar dose established that MOE-PS outperformed morpholino in the context of a severe SMA mouse model. The observation that MOE-PS ASO activity remains high not only in the liver, but also in all other examined peripheral tissues, one month following a high-dose of MOE10-29 suggests that ASO therapeutics can be used to treat a broad spectrum of diseases. Morpholino oligomers likely require higher doses and/or more frequent dosing to achieve comparable efficacy. As novel ASO-modification chemistries continue to emerge, a new modification or a combination of different chemistries could potentially result in even greater efficacy and tolerability for various applications.

## Supplementary Material

gkaa126_Supplemental_File

## References

[1] De Clercq E., Eckstein E., Merigan T.C. [Interferon induction increased through chemical modification of a synthetic polyribonucleotide]. Science (New York, N.Y.). 1969; 165:1137–1139. 10.1126/science.165.3898.1137 5801596

[2] Stein C.A., Subasinghe C., Shinozuka K., Cohen J.S. Physicochemical properties of phosphorothioate oligodeoxynucleotides. Nucleic Acids Res. 1988; 16:3209–3221. 2836790 10.1093/nar/16.8.3209 PMC336489

[3] Teplova M., Minasov G., Tereshko V., Inamati G.B., Cook P.D., Manoharan M., Egli M. Crystal structure and improved antisense properties of 2′-O-(2-methoxyethyl)-RNA. Nat. Struct. Biol. 1999; 6:535–539. 10360355 10.1038/9304

[4] Seth P.P., Siwkowski A., Allerson C.R., Vasquez G., Lee S., Prakash T.P., Kinberger G., Migawa M.T., Gaus H., Bhat B. et al. Design, synthesis and evaluation of constrained methoxyethyl (cMOE) and constrained ethyl (cEt) nucleoside analogs. Nucleic Acids Symp. Ser. 2008; 2004:553–554. 10.1093/nass/nrn280 18776499

[5] Kumar R., Singh S.K., Koshkin A.A., Rajwanshi V.K., Meldgaard M., Wengel J. The first analogues of LNA (locked nucleic acids): phosphorothioate-LNA and 2′-thio-LNA. Bioorg. Med. Chem. Lett. 1998; 8:2219–2222. 9873516 10.1016/s0960-894x(98)00366-7

[6] Hammond S.M., Hazell G., Shabanpoor F., Saleh A.F., Bowerman M., Sleigh J.N. Systemic peptide-mediated oligonucleotide therapy improves long-term survival in spinal muscular atrophy. Proc. Natl. Acad. Sci. U.S.A. 2016; 113:10962–10967. 27621445 10.1073/pnas.1605731113 PMC5047168

[7] Iversen P.L. Phosphorodiamidate morpholino oligomers: favorable properties for sequence-specific gene inactivation. Curr. Opin. Mol. Ther. 2001; 3:235–238. 11497346

[8] Stein C.A., Castanotto D. FDA-approved oligonucleotide therapies in 2017. Mol. Ther. 2017; 25:1069–1075. 28366767 10.1016/j.ymthe.2017.03.023 PMC5417833

[9] Scoto M., Finkel R., Mercuri E., Muntoni F. Genetic therapies for inherited neuromuscular disorders. Lancet Child Adolescent Health. 2018; 2:600–609. 30119719 10.1016/S2352-4642(18)30140-8

[10] Aartsma-Rus A. FDA approval of nusinersen for spinal muscular atrophy makes 2016 the year of splice modulating oligonucleotides. Nucleic Acid Ther. 2017; 27:67–69. 28346110 10.1089/nat.2017.0665

[11] Bennett C.F., Baker B.F., Pham N., Swayze E., Geary R.S. Pharmacology of antisense drugs. Annu. Rev. Pharmacol. Toxicol. 2017; 57:81–105. 27732800 10.1146/annurev-pharmtox-010716-104846

[12] Bennett C.F. Therapeutic antisense oligonucleotides are coming of age. Annu. Rev. Med. 2019; 70:307–321. 30691367 10.1146/annurev-med-041217-010829

[13] Syed Y.Y. Eteplirsen: first global approval. Drugs. 2016; 76:1699–1704. 27807823 10.1007/s40265-016-0657-1

[14] Ottesen E.W. ISS-N1 makes the first FDA-approved drug for spinal muscular atrophy. Transl. Neurosci. 2017; 8:1–6. 28400976 10.1515/tnsci-2017-0001 PMC5382937

[15] Wadman M. Antisense rescues babies from killer disease. Science. 2016; 354:1359–1360. 27980162 10.1126/science.354.6318.1359

[16] Pearn J. Classification of spinal muscular atrophies. Lancet (London, England). 1980; 1:919–922. 10.1016/s0140-6736(80)90847-8 6103267

[17] Crawford T.O., Pardo C.A. The neurobiology of childhood spinal muscular atrophy. Neurobiol. Dis. 1996; 3:97–110. 9173917 10.1006/nbdi.1996.0010

[18] Lefebvre S., Burglen L., Reboullet S., Clermont O., Burlet P., Viollet L., Benichou B., Cruaud C., Millasseau P., Zeviani M. et al. Identification and characterization of a spinal muscular atrophy-determining gene. Cell. 1995; 80:155–165. 7813012 10.1016/0092-8674(95)90460-3

[19] Burglen L., Spiegel R., Ignatius J., Cobben J.M., Landrieu P., Lefebvre S., Munnich A., Melki J. SMN gene deletion in variant of infantile spinal muscular atrophy. Lancet (London, England). 1995; 346:316–317. 10.1016/s0140-6736(95)92206-7 7630275

[20] Wu X., Wang S.H., Sun J., Krainer A.R., Hua Y., Prior T.W. A-44G transition in SMN2 intron 6 protects patients with spinal muscular atrophy. Hum. Mol. Genet. 2017; 26:2768–2780. 28460014 10.1093/hmg/ddx166 PMC5886194

[21] Lorson C.L., Hahnen E., Androphy E.J., Wirth B. A single nucleotide in the SMN gene regulates splicing and is responsible for spinal muscular atrophy. Proc. Natl. Acad. Sci. U.S.A. 1999; 96:6307–6311. 10339583 10.1073/pnas.96.11.6307 PMC26877

[22] Lefebvre S., Burlet P., Liu Q., Bertrandy S., Clermont O., Munnich A., Dreyfuss G., Melki J. Correlation between severity and SMN protein level in spinal muscular atrophy. Nat. Genet. 1997; 16:265–269. 9207792 10.1038/ng0797-265

[23] Hua Y., Vickers T.A., Baker B.F., Bennett C.F., Krainer A.R. Enhancement of SMN2 exon 7 inclusion by antisense oligonucleotides targeting the exon. PLoS Biol. 2007; 5:e73. 17355180 10.1371/journal.pbio.0050073 PMC1820610

[24] Hua Y., Vickers T.A., Okunola H.L., Bennett C.F., Krainer A.R. Antisense masking of an hnRNP A1/A2 intronic splicing silencer corrects SMN2 splicing in transgenic mice. Am. J. Hum. Genet. 2008; 82:834–848. 18371932 10.1016/j.ajhg.2008.01.014 PMC2427210

[25] Hua Y., Sahashi K., Rigo F., Hung G., Horev G., Bennett C.F., Krainer A.R. Peripheral SMN restoration is essential for long-term rescue of a severe spinal muscular atrophy mouse model. Nature. 2011; 478:123–126. 21979052 10.1038/nature10485 PMC3191865

[26] Hua Y., Liu Y.H., Sahashi K., Rigo F., Bennett C.F., Krainer A.R. Motor neuron cell-nonautonomous rescue of spinal muscular atrophy phenotypes in mild and severe transgenic mouse models. Genes Dev. 2015; 29:288–297. 25583329 10.1101/gad.256644.114 PMC4318145

[27] Passini M.A., Bu J., Richards A.M., Kinnecom C., Sardi S.P., Stanek L.M., Hua Y., Rigo F., Matson J., Hung G. et al. Antisense oligonucleotides delivered to the mouse CNS ameliorate symptoms of severe spinal muscular atrophy. Sci. Transl. Med. 2011; 3:72ra18. 10.1126/scitranslmed.3001777 PMC3140425 21368223

[28] Singh N.K., Singh N.N., Androphy E.J., Singh R.N. Splicing of a critical exon of human Survival Motor Neuron is regulated by a unique silencer element located in the last intron. Mol. Cell Biol. 2006; 26:1333–1346. 16449646 10.1128/MCB.26.4.1333-1346.2006 PMC1367187

[29] Finkel R.S., Chiriboga C.A., Vajsar J., Day J.W., Montes J., De Vivo D.C., Yamashita M., Rigo F., Hung G., Schneider E. et al. Treatment of infantile-onset spinal muscular atrophy with nusinersen: a phase 2, open-label, dose-escalation study. Lancet. 2016; 388:3017–3026. 27939059 10.1016/S0140-6736(16)31408-8

[30] Finkel R.S., Mercuri E., Darras B.T., Connolly A.M., Kuntz N.L., Kirschner J., Chiriboga C.A., Saito K., Servais L., Tizzano E. et al. Nusinersen versus sham control in infantile-onset spinal muscular atrophy. N. Engl. J. Med. 2017; 377:1723–1732. 29091570 10.1056/NEJMoa1702752

[31] Mercuri E., Darras B.T., Chiriboga C.A., Day J.W., Campbell C., Connolly A.M., Iannaccone S.T., Kirschner J., Kuntz N.L., Saito K. et al. Nusinersen versus sham control in later-onset spinal muscular atrophy. N. Engl. J. Med. 2018; 378:625–635. 29443664 10.1056/NEJMoa1710504

[32] Porensky P.N., Mitrpant C., McGovern V.L., Bevan A.K., Foust K.D., Kaspar B.K., Wilton S.D., Burghes A.H. A single administration of morpholino antisense oligomer rescues spinal muscular atrophy in mouse. Hum. Mol. Genet. 2012; 21:1625–1638. 22186025 10.1093/hmg/ddr600 PMC3298284

[33] Zhou H., Meng J., Marrosu E., Janghra N., Morgan J., Muntoni F. Repeated low doses of morpholino antisense oligomer: an intermediate mouse model of spinal muscular atrophy to explore the window of therapeutic response. Hum. Mol. Genet. 2015; 24:6265–6277. 26264577 10.1093/hmg/ddv329 PMC4614699

[34] Rigo F., Chun S.J., Norris D.A., Hung G., Lee S., Matson J., Fey R.A., Gaus H., Hua Y., Grundy J.S. et al. Pharmacology of a central nervous system delivered 2′-O-methoxyethyl-modified survival of motor neuron splicing oligonucleotide in mice and nonhuman primates. J. Pharmacol. Exp. Ther. 2014; 350:46–55. 24784568 10.1124/jpet.113.212407 PMC4056267

[35] Hsieh-Li H.M., Chang J.G., Jong Y.J., Wu M.H., Wang N.M., Tsai C.H., Li H. A mouse model for spinal muscular atrophy. Nat. Genet. 2000; 24:66–70. 10615130 10.1038/71709

[36] Foust K.D., Wang X., McGovern V.L., Braun L., Bevan A.K., Haidet A.M., Le T.T., Morales P.R., Rich M.M., Burghes A.H. et al. Rescue of the spinal muscular atrophy phenotype in a mouse model by early postnatal delivery of SMN. Nat. Biotechnol. 2010; 28:271–274. 20190738 10.1038/nbt.1610 PMC2889698

[37] Sheng L., Wan B., Feng P., Sun J., Rigo F., Bennett C.F., Akerman M., Krainer A.R., Hua Y. Downregulation of Survivin contributes to cell-cycle arrest during postnatal cardiac development in a severe spinal muscular atrophy mouse model. Hum. Mol. Genet. 2018; 27:486–498. 29220503 10.1093/hmg/ddx418 PMC5886172

[38] Maxwell G.K., Szunyogova E., Shorrock H.K., Gillingwater T.H. Developmental and degenerative cardiac defects in the Taiwanese mouse model of severe spinal muscular atrophy. J. Anat. 2018; 232:965–978. 29473159 10.1111/joa.12793 PMC5978979

[39] Szunyogova E., Zhou H., Maxwell G.K., Powis R.A., Muntoni F., Gillingwater T.H., Parson S.H. Survival Motor Neuron (SMN) protein is required for normal mouse liver development. Sci. Rep. 2016; 6:34635. 27698380 10.1038/srep34635 PMC5048144

[40] Khairallah M.T., Astroski J., Custer S.K., Androphy E.J., Franklin C.L., Lorson C.L. SMN deficiency negatively impacts red pulp macrophages and spleen development in mouse models of spinal muscular atrophy. Hum. Mol. Genet. 2017; 26:932–941. 28062667 10.1093/hmg/ddx008 PMC6075362

[41] Liu Q., Dreyfuss G. A novel nuclear structure containing the survival of motor neurons protein. EMBO J. 1996; 15:3555–3565. 8670859 PMC451956

[42] Coovert D.D., Le T.T., McAndrew P.E., Strasswimmer J., Crawford T.O., Mendell J.R., Coulson S.E., Androphy E.J., Prior T.W., Burghes A.H. The survival motor neuron protein in spinal muscular atrophy. Hum. Mol. Genet. 1997; 6:1205–1214. 9259265 10.1093/hmg/6.8.1205

[43] Ling K.K., Gibbs R.M., Feng Z., Ko C.P. Severe neuromuscular denervation of clinically relevant muscles in a mouse model of spinal muscular atrophy. Hum. Mol. Genet. 2012; 21:185–195. 21968514 10.1093/hmg/ddr453 PMC3235013

[44] Lin T.L., Chen T.H., Hsu Y.Y., Cheng Y.H., Juang B.T., Jong Y.J. Selective neuromuscular denervation in Taiwanese severe SMA mouse can be reversed by morpholino antisense oligonucleotides. PLoS One. 2016; 11:e0154723. 27124114 10.1371/journal.pone.0154723 PMC4849667

[45] Southwell A.L., Skotte N.H., Bennett C.F., Hayden M.R. Antisense oligonucleotide therapeutics for inherited neurodegenerative diseases. Trends Mol. Med. 2012; 18:634–643. 23026741 10.1016/j.molmed.2012.09.001

[46] Wurster C.D., Ludolph A.C. Antisense oligonucleotides in neurological disorders. Ther. Adv. Neurol. Disord. 2018; 11:1756286418776932. 29854003 10.1177/1756286418776932 PMC5971383

[47] Levin A.A. Treating disease at the RNA level with oligonucleotides. N. Engl. J. Med. 2019; 380:57–70. 30601736 10.1056/NEJMra1705346

[48] Sazani P., Gemignani F., Kang S.H., Maier M.A., Manoharan M., Persmark M., Bortner D., Kole R. Systemically delivered antisense oligomers upregulate gene expression in mouse tissues. Nat. Biotechnol. 2002; 20:1228–1233. 12426578 10.1038/nbt759

[49] Roberts J., Palma E., Sazani P., Orum H., Cho M., Kole R. Efficient and persistent splice switching by systemically delivered LNA oligonucleotides in mice. Mol. Ther. 2006; 14:471–475. 16854630 10.1016/j.ymthe.2006.05.017

[50] Prakash T.P., Yu J., Kinberger G.A., Low A., Jackson M., Rigo F., Swayze E.E., Seth P.P. Evaluation of the effect of 2′-O-methyl, fluoro hexitol, bicyclo and Morpholino nucleic acid modifications on potency of GalNAc conjugated antisense oligonucleotides in mice. Bioorg. Med. Chem. Lett. 2018; 28:3774–3779. 30342955 10.1016/j.bmcl.2018.10.011

[51] Shen W., Liang X.H., Crooke S.T. Phosphorothioate oligonucleotides can displace NEAT1 RNA and form nuclear paraspeckle-like structures. Nucleic. Acids. Res. 2014; 42:8648–8662. 25013176 10.1093/nar/gku579 PMC4117792

[52] Wang Y., Shen W., Liang X.H., Crooke S.T. Phosphorothioate antisense oligonucleotides bind P-body proteins and mediate P-body assembly. Nucleic Acid Ther. 2019; 29:343–358. 31429620 10.1089/nat.2019.0806

[53] Hua Y., Sahashi K., Hung G., Rigo F., Passini M.A., Bennett C.F., Krainer A.R. Antisense correction of SMN2 splicing in the CNS rescues necrosis in a type III SMA mouse model. Genes Dev. 2010; 24:1634–1644. 20624852 10.1101/gad.1941310 PMC2912561

[54] Rigo F., Hua Y., Chun S.J., Prakash T.P., Krainer A.R., Bennett C.F. Synthetic oligonucleotides recruit ILF2/3 to RNA transcripts to modulate splicing. Nat. Chem. Biol. 2012; 8:555–561. 22504300 10.1038/nchembio.939 PMC5021312

[55] Williams J.H., Schray R.C., Patterson C.A., Ayitey S.O., Tallent M.K., Lutz G.J. Oligonucleotide-mediated survival of motor neuron protein expression in CNS improves phenotype in a mouse model of spinal muscular atrophy. J. Neurosci. 2009; 29:7633–7638. 19535574 10.1523/JNEUROSCI.0950-09.2009 PMC6665627

[56] Robin V., Griffith G., Carter J.L., Leumann C.J., Garcia L., Goyenvalle A. Efficient SMN rescue following subcutaneous Tricyclo-DNA antisense oligonucleotide treatment. Mol. Ther. Nucleic Acids. 2017; 7:81–89. 28624227 10.1016/j.omtn.2017.02.009 PMC5415958

