# Supplementary material for: Comparison of the efficacy of MOE and PMO modifications of systemic antisense oligonucleotides in a severe SMA mouse model
Source: Nucleic Acids Res. 2020 Feb 27;48(6):2853–65. doi: 10.1093/nar/gkaa126 (PMC7102994; doi:10.1093/nar/gkaa126)
Supplement: gkaa126_Supplemental_File [file gkaa126_supplemental_file.pdf]

Sheng_Supplemental Fig. 1

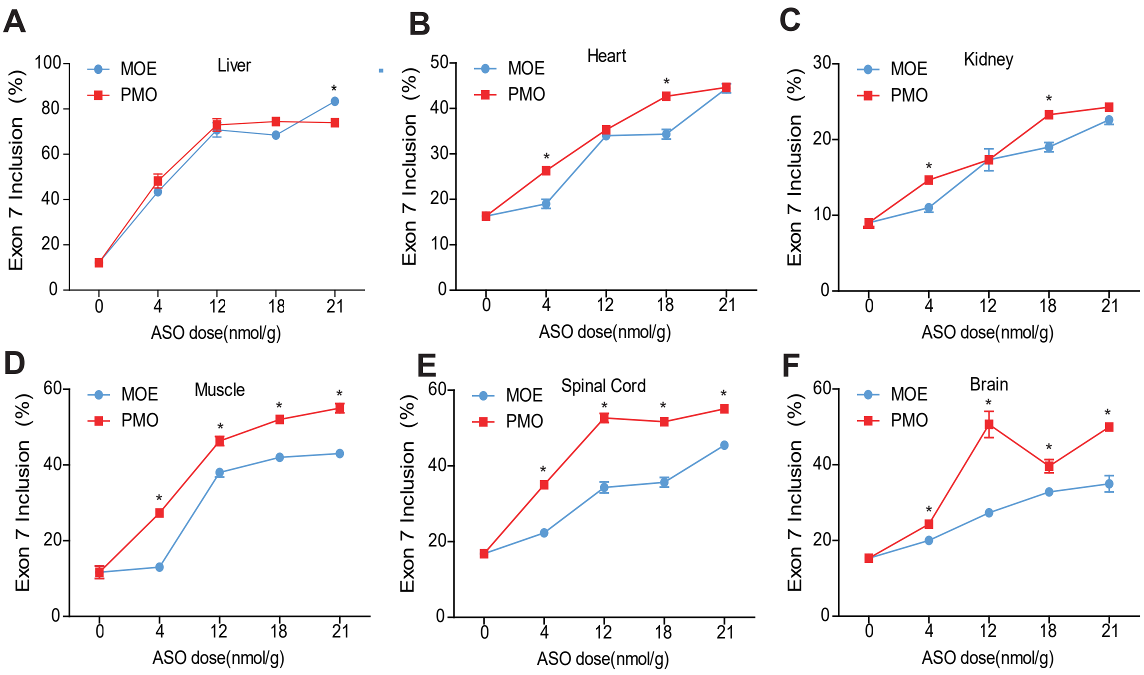

Fig S1 SMN2 splicing changes at P7 in mouse peripheral and CNS tissues after SC injection with four different doses (4, 12, 18, 21 nmol/g) of MOE and PMO1 ASO10-29. The exon 7 inclusion of the higher dose (18 and 21 nmol/g) groups had little difference change compared to 12 nmol/g. In PMO treated groups, the exon 7 inclusion of muscle, spinal cord and brain are significantly higher than that of MOE groups at four different doses.

Sheng_Supplemental Fig. 2

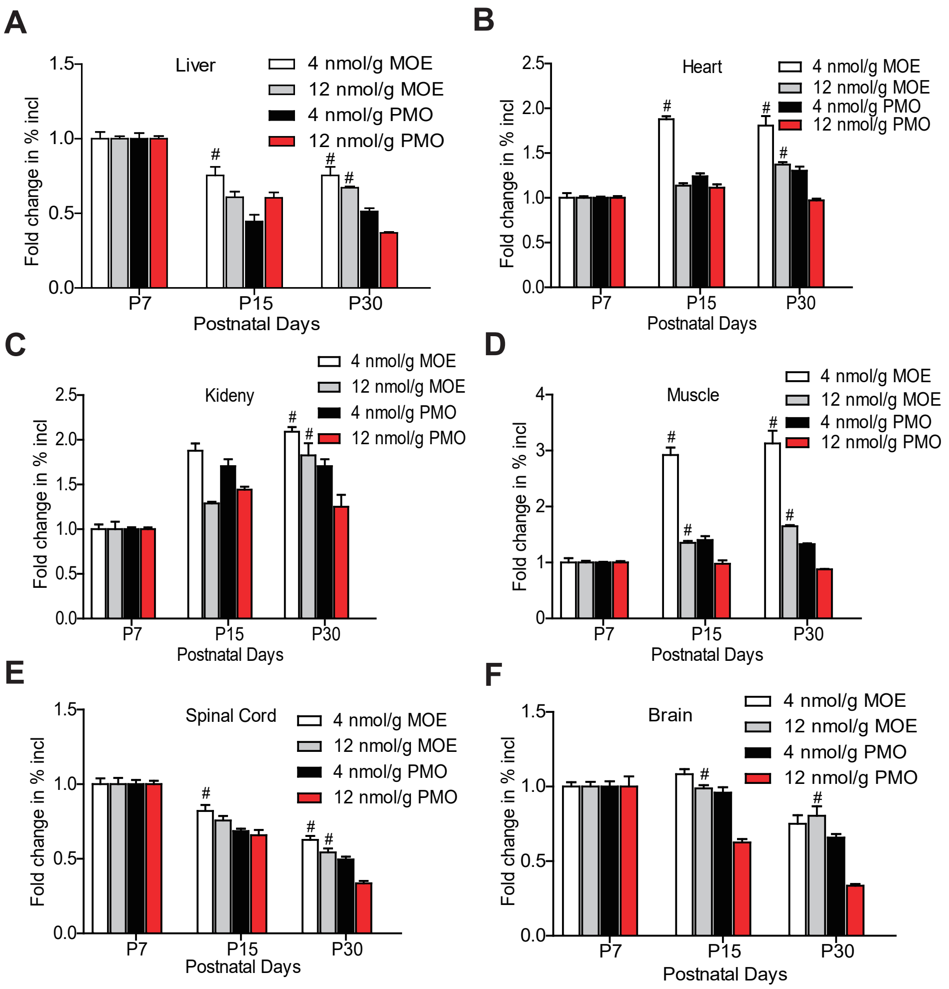

Fig S2 Change fold of exon 7 inclusion of P15 and P30 compared to P7 after SC injection of MOE and PMO ASO10-29 in mouse peripheral and CNS tissues.
Change fold=(Exon 7 inclusion of P15/30-Exon 7 inclusion of P7)/Exon 7 inclusion of P7.
(A,E,F) The exon 7 inclusion of PMO groups decreased rapidly in liver, spinal cord and brain after P7, compared to MOE. (B-D) The exon 7 inclsion of MOE groups increased in heart, kidney and muscle after P7, suggesting the effect of MOE sustained longer time.
(#) P <0.05 versus the same dose of different group.

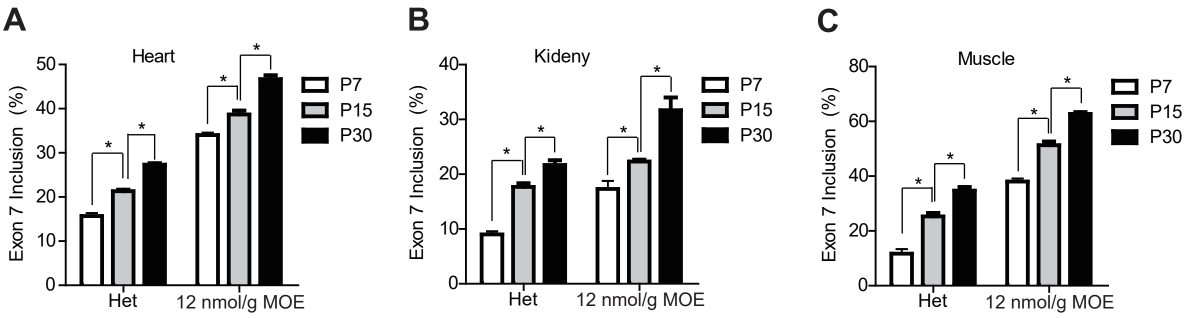

Fig S3 Exon 7 inclusion of P15 versus P7, and P30 versus P15 after SC injection of high dose-MOE ASO10-29 treated mice and heterozygous mice in heart, kidney and muscle tissues.

(A-C) the percentage of exon7 inclusion in the heart, kidneys and skeletal muscles of mice treated with high-dose MOE10-29 and heterozygous mice increased over time during the first postnatal monthas (*) P <0.05.

Sheng_Supplemental Fig. 4

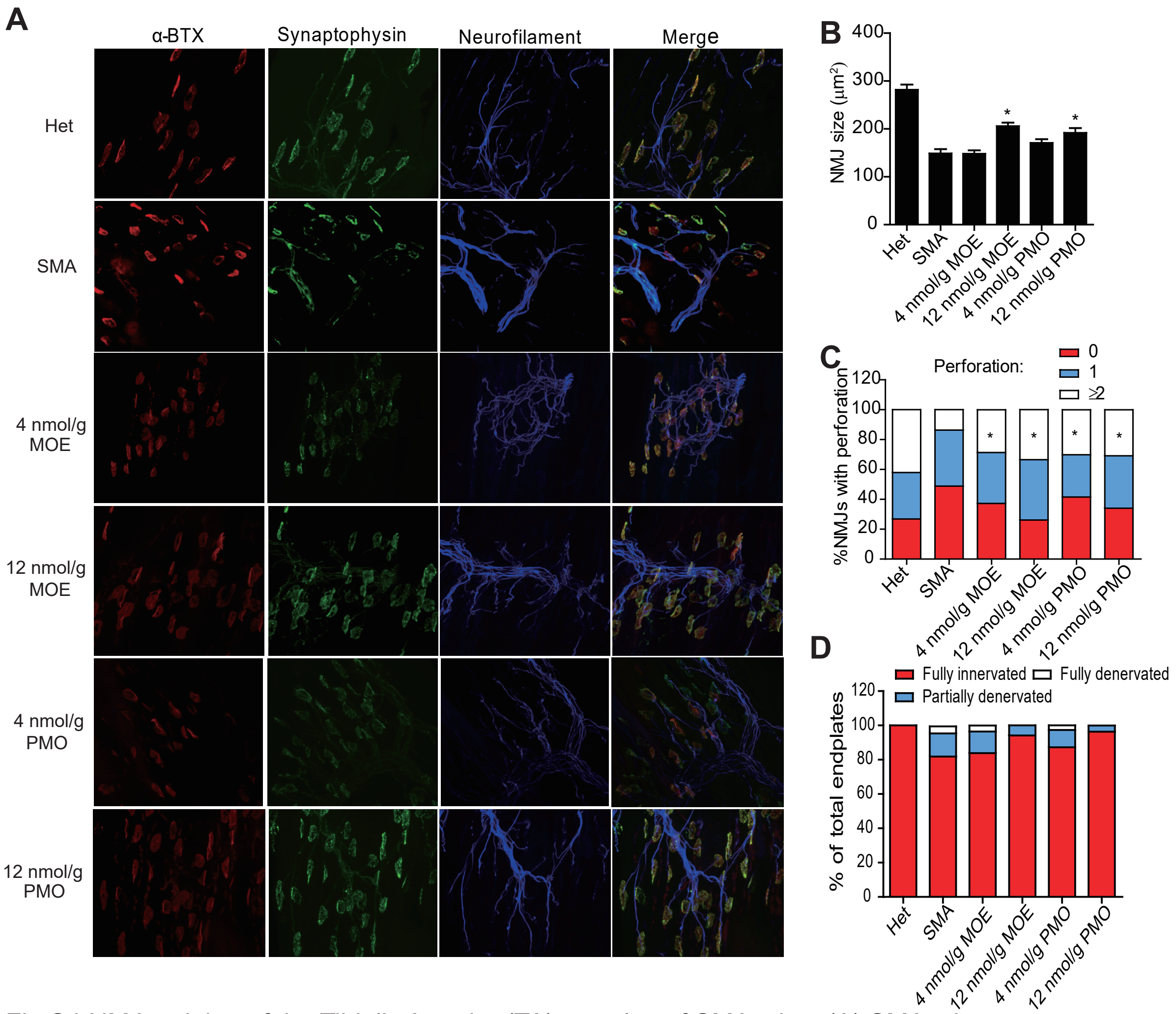

Fig S4 NMJ staining of the Tibialis Anterior (TA) muscles of SMA mice. (A) SMA mice were treated with saline, MOE and morpholino ASO10-29. Untreated heterozygous mice (Het, n = 4) were used as normal controls. Mice were treated on P0 and P1 and TA were collected on P9 and stained for neurofilament with anti-neurofilament (blue), nerve terminals with anti-synaptophysin (green), andmotor endplates with a-bungarotoxin (α-BTX, red). (B) NMJ area was measured from (A) (4 mice per group and 3 counts per mouse). After treatment with MOE10-29 or PMO10-29, the endplate size was increased by 0% (low MOE), 38% (high MOE), 14% (low PMO) and 28% (high PMO), respectively, compared to severe SMA mice. (C) Quantification of perforations number identified by α-BTX, as shown in (A) (4 mice per group and 3counts per mouse). and the percentage of NMJ containing ≥2 perforations was increased by 2 fold, 2.4 fold, 2.2 fold and 2.3 fold, respectively, compared to severe SMA mice. (D) Percentage of innervated endplates (red), partially denervated endplates (blue), and fully denervated endplates (white) in total endplate numbers were analyzed from (A) (4 mice per group and 3 counts per mouse). There is no significant difference between heterozygous mice and SMA mice in percentage of fully and partially denervated NMJ. (*) $P < 0.05$ versus the low dose of same group;(#) $P < 0.05$ versus the same dose of different group.

Sheng_supplemental Fig.5

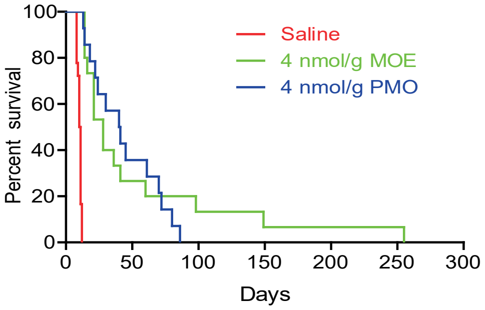

Fig S5 Intracerebroventricular (ICV) administration of MOE and morpholino ASO10-29 modestly extend survival time of SMA mice. Litters were given two injections between P0 and P1 with 4 nmol/g/injection. Each group had 12 mice. MOE and morpholino 10-29 increased the median and mean survivals to 28 and 54 days, 41 and 44 days, respectively. There was no significant difference between MOE and morpholino groups.
